# Insight on Glucose and Fructose Absorption and Relevance in the Enterocyte Milieu

**DOI:** 10.3390/nu14030517

**Published:** 2022-01-25

**Authors:** Elena Chiarello, Mattia Di Nunzio, Gianfranco Picone, Giorgia Antonelli, Francesco Capozzi, Alessandra Bordoni

**Affiliations:** 1 Department of Agri-Food Sciences and Technologies (DISTAL), University of Bologna, Piazza Goidanich 60, 47521 Cesena, Italy; elena.chiarello2@unibo.it (E.C.); gianfranco.picone@unibo.it (G.P.); giorgia.antonelli4@unibo.it (G.A.); francesco.capozzi@unibo.it (F.C.); 2 Department of Food, Environmental and Nutritional Sciences (Defens), University of Milan, via Celoria 2, 20133 Milan, Italy; mattia.dinunzio@unimi.it; 3 Interdepartmental Centre for Industrial Agri-Food Research (CIRI), University of Bologna, Piazza Goidanich 60, 47521 Cesena, Italy

**Keywords:** fructose, glucose, enterocytes

## Abstract

Although epidemiological studies indicate a strong correlation between high sugar intake and metabolic diseases, the biological mechanisms underlying this link are still controversial. To further examine the modification and crosstalk occurring in enterocyte metabolism during sugar absorption, in this study we evaluate the diffusion and intestinal metabolism of glucose, fructose and sucrose, which were supplemented in equimolar concentration to Caco-2 cells grown on polyester membrane inserts. At different time points after supplementation, changes in metabolite concentration were evaluated in the apical and basolateral chambers by nuclear magnetic resonance (NMR) and gas-chromatography (GC). Sucrose was only minimally hydrolyzed by Caco-2 cells. Upon supplementation, we observed a faster uptake of fructose than glucose, the pentose sugar being also faster catabolized. Monosaccharide absorption was concomitant to the synthesis/transport of other metabolites, which occurred differently in glucose and fructose supplemented cells. Our results confirm the prominent role of intestinal cells in fructose metabolism and clearance after absorption, representing a further step forward in the understanding of the role of dietary sugars. Future research, including targeted analysis on specific transporters/enzymes and the use of labeled substrates, will be helpful to confirm the present results and their interpretation.

## 1. Introduction

Glucose (GLU) and fructose (FRU) are the main monosaccharides in the human diet. FRU and GLU share the same molecular formula (C_6_H_12_O_6_) and energy value (4 kcal/g), but have different sweetening power and glycemic index [1], satiating capacity [2], absorption mechanism [3], and metabolism not only once absorbed, but also within the intestinal cell [4]. Sucrose (SUC), a disaccharide composed of GLU and FRU, is one of the highest dietary sources of FRU in addition to corn/maple syrup, honey, molasses, fruit and fruit juices.

GLU is the main catabolic and anabolic cell substrate that controls energy homeostasis in the human body. The classical mechanism for intestinal glucose absorption involves uptake across the apical membrane of enterocytes via sodium-glucose co-transporter 1 (SGLT1). However, it has been recognized that the apical uptake phase has two distinct elements: a saturable, phloridzin-sensitive fraction (SGLT1) and a diffusive component, suggesting that more than one transporter may be involved. Studies have revealed that glucose transporter 2 (GLUT2) is also expressed at the apical membrane of enterocytes during the digestive phase and can contribute significantly to glucose absorption [5]. The release of glucose into the circulation is mediated by GLUT2 located on the basolateral surface of enterocytes [6]. GLUT2 is also responsible for the efflux of FRU, which enters the intestinal cells mainly through the GLUT5 (SLC2A5) transporter.

Although epidemiological studies indicate a strong correlation between high sugar intake and metabolic diseases, the biological mechanisms underlying this link are still controversial [7], and the role of the gastro-enteric tract is often neglected. To exert their effect in the human body, sugars must be released from the food matrix, eventually hydrolyzed into monosaccharides, and absorbed. Despite the modulation of sugar transporters having been deeply investigated [8,9], the metabolic modifications occurring in the enterocyte during absorption and the impact of the monosaccharides on the biochemical events taking place within the intestinal cell are still poorly understood.

The aim of the present study is to further examine the crosstalk occurring in enterocyte during sugar absorption, giving a dynamic, untargeted, and omni-comprehensive vision of the events. We use Caco-2 cells grown on trans well inserts as model system. Caco-2 cells, a human intestinal cell line derived from colon cancer, differentiate on the membrane filter in trans well culture into cells that exhibit both morphological and biochemical features characteristic of intestinal epithelial cells. When grown on filter supports, after reaching confluence, the Caco-2 cells spontaneously start to differentiate into a polarized cell layer with apical microvilli and intercellular tight junction complexes [10]. The Caco-2 cell model has been extensively utilized for studies on sugar transport as a model for the small intestine, since the expression and membrane location of GLUTs and SGLT1 are well known under a wide variety of conditions. In addition, the pathways for utilization of FRU are intact since fructose can be readily used as an energy source by Caco-2 cells [11,12].

Cells were supplemented with equimolar concentrations of GLU, FRU or SUC. The concentration chosen for supplementation corresponds to the physiological luminal GLU concentration in the small intestine under normal conditions [13]. To avoid bias, the possible cytotoxicity of supplemented sugars was also evaluated.

The changes in metabolite concentration occurring in the apical (AC) and basolateral chambers (BLC) were evaluated by 1H-NMR spectroscopy and gas-chromatography (GC), allowing us to follow in an 8 h timeframe the overall metabolic response of enterocytes to the exposure to GLU, FRU and SUC.

## 2. Materials and Methods

### 2.1. Chemicals

Dulbecco’s modified Eagle’s medium (DMEM), penicillin, streptomycin, and Dulbecco’s phosphate-buffered saline (DPBS) were purchased from Lonza (Basel, Switzerland). All other chemicals and solvents were of the highest analytical grade from Sigma-Aldrich Co. (St. Louis, MO, USA).

### 2.2. Cell Culture and Supplementation

Experiments were carried out using Caco-2 cells (European Collection of Authenticated Cell Cultures (ECACC) 86010202, passage number between 50–60) seeded on a polyester (PET) membrane insert (Trans well; 0.4 μm pore size, 2 × 10^6^ pores/cm^2^, 4.2 cm^2^ insert membrane growth area) (Corning Incorporated, New York, NY, USA). Cells were seeded at the density of 2 × 10^5^ cells/well and grown for 21 days in DMEM supplemented with fetal bovine serum (FBS) (10% *v*/*v*), non-essential amino acids (1% *v*/*v*), 100 U/mL penicillin and 100 μg/mL streptomycin. After 21 days of culture, the Caco-2 cells appeared completely differentiated and polarized, resembling the morphological and functional features of the mature enterocytes.

Before carrying out the experiments, the monolayer integrity was assessed by measuring the trans-epithelial electric resistance of the cell monolayer using the Millicell ERS apparatus (Millipore, Burlington, MA, USA). TEER values between 1000 and 2000 Ω*cm^2^ were considered acceptable for the transport assay [14].

Before sugar supplementation, apical and basal compartments were washed twice with 1 mL phosphate buffer solution (PBS), and then filled with DMEM (2 mL) containing 25 mM GLU or FRU or SUC, without PBS and not essential amino acid (apical chamber) or with the same volume of DPBS (basolateral chamber).

After 0.5, 1, 2, 4, 6 or 8 h incubation, cell cytotoxicity was assessed, and media were collected from both chambers to perform ^1^H-NMR and GC analyses.

### 2.3. Cytotoxicity Evaluation

Cytotoxicity was evaluated by the methylthiazolyldiphenyl-tetrazolium bromide (MTT) assay and microscopic examination:

(a) Viability was determined by conversion of the MTT salt to its formazan product detected at 560 nm using a Tecan Infinite M200 microplate reader (Tecan, Männedorf, Switzerland) [15]. Cell viability was expressed as percentage of viability in control cells, assigned as 100%.
(b) Light microscopy examination of cell morphology and monolayer integrity was performed using an inverted confocal light microscopy model IB (Exacta Optech, Modena, Italy) using 10×, 25× and 40× as magnification [16].

### 2.4. HR ^1^H-NMR

A total of 500 μL of DMEM or DPBS were added to 10 μL of a D2O solution of 100 mM 2.2-dimethyl-2-silapentane-d6-5-sulfonic (d6-DSS) with a final concentration in the NMR tube of 0.58 mM. H-NMR spectra were recorded at 298 K on a Bruker US+ Avance III spectrometer operating at 600 MHz, equipped with a BBI-z probe and a B-ACS 60 sampler for automation (Bruker BioSpin, Karlsruhe, Germany). The monodeuterated water (HOD) residual signal was suppressed by applying the noesygppr1d.comp pulse sequence with pre-saturation during relaxation delay and mixing time and spoil gradient. Each spectrum was acquired using 32 K data points over a 7,194,245 Hz spectral width (12 ppm) and adding 256 transients. A recycle delay of 5 s and a 90° pulse of 11.190 μs were set up. Acquisition time (2.277 s) and recycle delay were adjusted to be 5 times longer than the longitudinal relaxation time of the protons under investigation, which was not longer than 1.4. The data were Fourier transformed and phase and baseline corrections were automatically performed using TopSpin version 3.5pl (Bruker BioSpin, Karlsruhe, Germany). Signals were identified by comparing their chemical shift and multiplicity with the Chenomx Profiler software data bank (ver. 8.1, Edmonton, AB, Canada) and data in the literature [17].

### 2.5. Lipid Extraction and Fatty Acid Composition Analysis

Total lipids in Caco-2 cells, AC and BLC were extracted, and relative fatty acids methylated as previously described [18]. Before methylation, pentadecanoic acid was added as internal standard. The qualitative and quantitative content of fatty acid methyl esters (FAMEs) was determined by fast-GC (GC-2030AF; Shimadzu, Kyoto, Japan) using a capillary column (30 mt, 0.2 μm film thickness) with a programed temperature gradient (50–250 °C, 10 °C/min). The gas chromatographic peaks were identified based on their retention time ratios relative to methyl stearate and predetermined by use of authentic samples [19]. Gas chromatographic traces and quantitative evaluations were obtained using a Lab Solution software (Shimadzu, Kyoto, Japan).

### 2.6. Statistical Analysis

All data are means ± standard deviation (SD) of at least 3 samples from independent experiments. In each experimental condition, metabolite concentration at different time points was statistically evaluated by the one-way analysis of variance (ANOVA) followed by Tukey’s post hoc test. To compare the effect of GLU and FRU supplementation at the same time point, we used an unpaired *t*-test considering *p* < 0.05 as significant.

## 3. Results

### 3.1. Cytotoxicity

In all conditions, MTT assay and light microscopy examination did not evidence either modification in cell viability or in monolayer integrity at any time point (data not shown).

### 3.2. Metabolite Diffusion

**Figure 1:**
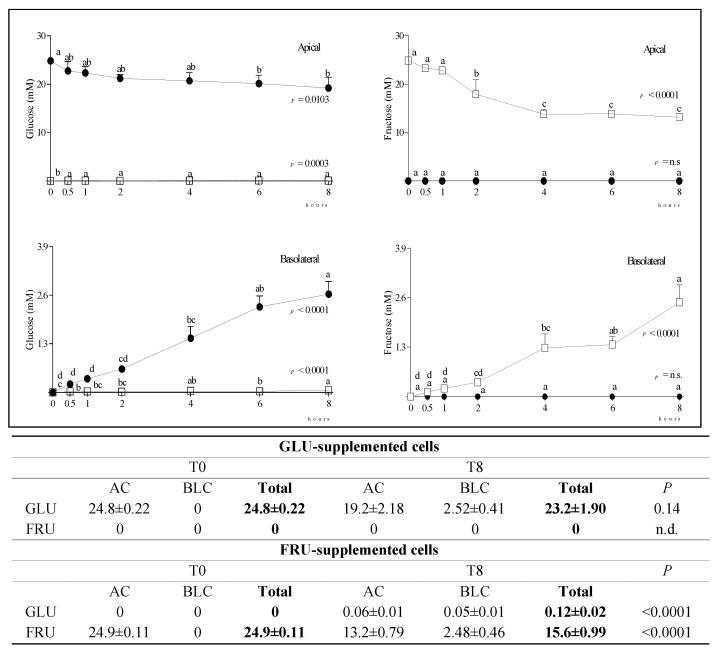
Changes in GLU and FRU concentration in the AC and BLC of GLU- and FRU-supplemented cells. Data are means ± SD of at least 3 samples from independent experiments. In the graphs, GLU (**left panel**) and FRU (**right panel**) concentrations (mM) at different time points are indicated by black circles (GLU-supplemented cells) or white squares (FRU-supplemented cells). In each experimental condition, differences between different time points were evaluated by the one-way ANOVA with Tukey’s post hoc test (different letters indicate significant differences). In the table, the total concentration of the monosaccharides at T0 and T8 in the same experimental condition is reported and compared by unpaired *t*-test. In each condition, statistical analysis was by an unpaired *t*-test to evaluate differences between T0 and T8. GLU: glucose; FRU: fructose; AC: apical chamber; BLC: basolateral chamber.

The time course and extent of GLU and FRU diffusion from the AC to the BLC in GLU and FRU-supplemented cells are reported in Figure 1. Although in both conditions the reduction of the concentration of the monosaccharide supplemented in the AC was concomitant to its increase in the BLC, in GLU-supplemented cells the total amount (AC + BLC) of GLU was similar at T0 and T8, while in FRU-supplemented cells the total amount of FRU was significantly lower at T8 than T0, which indicates the active metabolization of the supplemented ketohexose, including its conversion in small amount to aldohexose.

**Figure 2:**
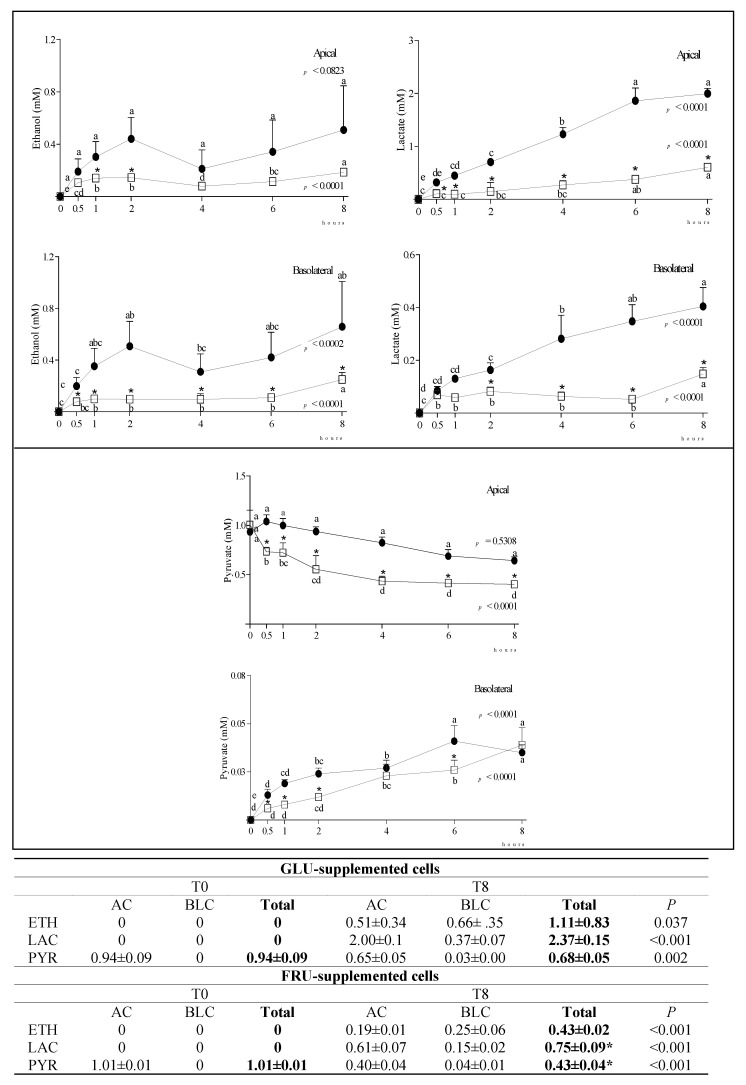
Changes in ETH, LAC and PYR concentration in the AC and BLC of GLU- and FRU-supplemented cells. Data are means ± SD of at least 3 samples from independent experiments. In the graphs, carbonyl metabolites concentration (mM) at different time points is indicated by black circles (GLU-supplemented cells) or white squares (FRU-supplemented cells). In each experimental condition, differences between different time points were evaluated by the one-way ANOVA with Tukey’s post hoc test (different letters indicate significant differences). At each time point, differences between GLU- and FRU-supplemented cells were evaluated by unpaired *t*-test (* at least *p* < 0.05). In the table, the total concentration of metabolites at T0 and T8 in the same experimental condition is reported and compared by unpaired t test. Statistical differences between the two experimental conditions in the metabolite total concentration at T8 were calculated by an unpaired *t*-test (* *p* at least < 0.05). GLU: glucose; FRU: fructose; AC: apical chamber; BLC: basolateral chamber; ETH: ethanol; LAC: lactate; PYR: pyruvate.

In SUC-supplemented cells, we detected very low amounts of GLU or FRU (<0.65 mM) either in the AC or BLC at any time after supplementation, indicating that the disaccharide was only minimally hydrolyzed. At longer incubation times, SUC slightly diffused from the AC to the BLC, probably via paracellular transport (Supplementary Figure S1). Since SUC-supplemented cells did not reproduce a physiological condition, we did not focus on this condition. Regardless of this, the obtained results in SUC-supplemented cells are provided as Supplementary Material (Supplementary Figures S1–S6). The time-course of changes in carbonyl metabolite (ethanol—ETH, lactate—LAC, pyruvate—PYR) concentration in the AC and BLC of GLU- and FRU-supplemented cells, and their total concentration (AC + BLC) at T0 and T8, is reported in Figure 2. Although ETH and LAC were not present in the supplemented media, their concentration significantly increased with time in both chambers, more evidently in GLU-supplemented cells. In these cells, PYR concentration in the AC was almost constant over time, while it significantly decreased in FRU supplemented cells. Notwithstanding, at T8, PYR concentration in the BLC was similar in both conditions. The total amount (AC + BLC) of PYR was significantly lower at T8 than T0, indicating its active metabolization particularly in FRU-supplemented cells.

**Figure 3:**
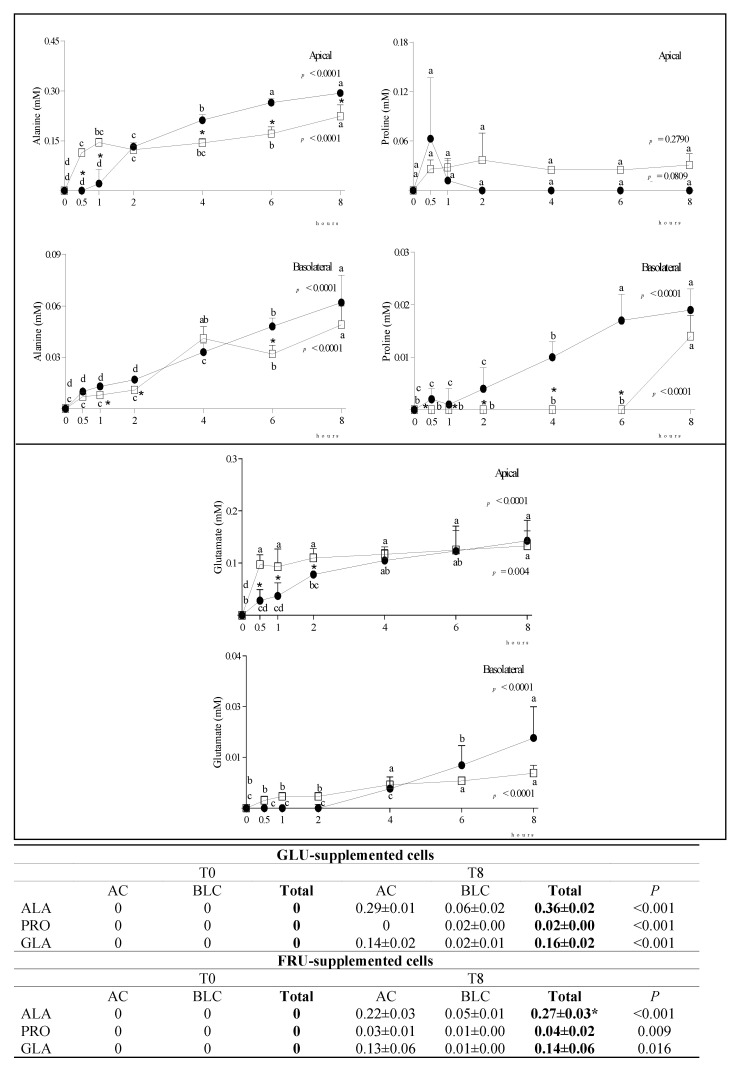
Changes in ALA, PRO and GLA concentration in the AC and BLC of GLU- and FRU-supplemented cells. Data are means ± SD of at least 3 samples from independent experiments. In graphs, amino acid concentration (mM) at different time points is indicated by black circles (GLU-supplemented cells) or white squares (FRU-supplemented cells). In each experimental condition, differences between different time points were evaluated by the one-way ANOVA with Tukey’s post hoc test (different letters indicate significant differences). At each time point, differences between GLU- and FRU-supplemented cells were evaluated by unpaired *t*-test (* at least *p* < 0.05). In the table, the total concentration of metabolites at T0 and T8 in the same experimental condition is reported and compared by an unpaired *t*-test. Statistical differences between the two experimental conditions in the metabolite total concentration at T8 were calculated by an unpaired *t*-test (* *p* at least < 0.05). GLU: glucose; FRU: fructose; AC: apical chamber; BLC: basolateral chamber; ALA: alanine; PRO: proline; GLA: glutamate.

At T0, alanine (ALA), proline (PRO) and glutamic acid (GLA) were not present either in the AC or in the BLC medium. Over time, their concentration significantly increased in both chambers (Figure 3).

Glutamine (GLN), phenylalanine (PHE), leucine (LEU), isoleucine (ILE), valine (VAL), methionine (MET), threonine (THR), lysine (LYS), arginine (ARG), serine (SER) and tyrosine (TYR) were already present in the media supplemented in the AC at concentration ranging from about 1.8 mM (GLN) to 0.2 mM (MET), while their concentration was null in the BLC medium. In GLU-supplemented cells, these amino acids diffused to the BLC, and the total (AC + BLC) amount of the most of them was similar at T0 and T8. Conversely, in FRU-supplemented cells, the total concentration of all the above listed amino acids except GLN and THR was significantly lower at T8 than T0, suggesting that the overtime decrease in their concentration in the AC was due to a metabolic conversion rather than simply a diffusion toward the BLC (Supplementary Figures S7 and S8).

Finally, to verify whether and to which extent the supplemented GLU and FRU, and eventually other substrates, were converted to acetyl-CoA providing carbon inputs into fatty acids, we compared the fatty acid content in cells, AC and BC, at T0 and T8 in the two experimental conditions. At T8, cell total FAME content was higher than at T0, mainly due to an increase in saturated and monounsaturated fatty acid concentration, without any significant difference between GLU and FRU supplementation (Table 1).

At T0, media in the AC and BLC did not contain any fatty acid. After 8 h of supplementation, myristic, palmitic, stearic and oleic acids were detected in both chambers, palmitic and stearic acid concentration being higher in the BLC of FRU than GLU supplemented cells (Table 2). Summing the fatty acid content in cells, AC, and BLC, at T8, a significant difference was detected between GLU- (total FAME = 228.8 ± 7.7 µg/well) and FRU-supplemented cells (total FAME = 265.2 ± 18.7 µg/well; *p* = 0.0356).

## 4. Discussion

After the supplementation of Caco-2 cells with equimolar amounts of GLU or FRU, we observed a faster reduction of the ketohexose than the aldohexose sugar concentration in the AC. This more efficient uptake could be related to the increased GLUT5 mRNA stability, already observed in human Caco-2 cells after FRU exposure [20], or to the overexpression of GLUT5 mRNA and protein by dietary FRU [3]. Notwithstanding the more efficient diffusion of FRU than GLU, at T8, the BLC concentration of the supplemented monosaccharide was similar in the two experimental conditions, suggesting that FRU was only partially released in the BLC in its parent form, and was it actively metabolized within the cell. Indeed, in GLU-supplemented cells the total concentration (AC + BLC) of GLU was similar at T0 and T8, while in FRU-supplemented cells, FRU total concentration at T8 was significantly lower than at T0.

SLGT1 functionality was reported as different in Caco-2 cells obtained from three different cell banks, being lower in cells from ECACC than Leibniz Institute DSMZ-German Collection of Microorganisms and Cell Cultures (DSMZ) [21]. In that work, the values obtained in Caco-2 cells were not compared to in vivo SLGT1 functionality, so observed differences did not mirror the reliability of the different cell lines, but simply a biological variability. In addition, Steffenson et al. evaluated the functionality of SGLT1 and not of sodium-independent transporters, such as GLUT2, and Alzaid et al. [22], using ^3^H-D-glucose as tracer, revealed that the diffusive component accounts for more than 40% of total glucose uptake in Caco-2 cells. Therefore, a lower SLGT1 functionality in our cell line, if any, had a limited effect on total GLU transport.

As reported above, we supplemented cells with the same concentration (25 mM) of GLU and FRU. This concentration corresponds to the physiological luminal GLU concentration in the small intestine under normal conditions. Although in the literature there are not specific data on intestinal FRU concentrations under normal conditions, we speculated that a 25 mM concentration of FRU could correspond to a high FRU intake. Intestinal FRU catabolism is dose dependent, and at low doses, the 90% of FRU is cleared by the intestine [23]. Conversely, Patel et al. [24] estimated that as much as 10–30% of an absorbed FRU load may undergo catabolism within enterocytes. In our study, about 40% of the supplemented ketohexose sugar was actively catabolized by Caco-2 cells, confirming that we were reproducing a condition that is considered risky for the development of metabolic diseases.

Fructolysis leads to increased glyceraldehyde, dihydroxy-acetone-phosphate, and glyceraldehyde-3-phosphate production, which are the sources of gluconeogenic and lipogenic substrates. In FRU-supplemented cells, a small amount of GLU was detected, in agreement with the reported existence of a conversion of FRU to GLU in human jejunum [25]. In GLU-supplemented cells, the conversion of GLU to FRU was not evidenced, confirming that FRU biosynthesis from GLU is mostly inactive under physiological conditions [4].

In agreement with results already obtained in mice [23], in our experimental conditions, LAC formation was greater for GLU than FRU, which was poorly converted. LAC was detected in both chambers, consistent with the presence of the main intestinal LAC transporter both in the apical and basolateral membrane of the enterocyte [26]. Interestingly, a small amount of ETH was detected in both chambers. The ETH formation inside human tissues was considered in the past—the so-called ‘auto-brewery’ syndrome [27]—and it was mainly ascribed to the microbial fermentation of the carbohydrates in the gastro-intestinal tract. In addition to the synthesis of ETH by the intestinal flora, some researchers have considered the possibility of ETH formation inside human tissues [28]. Our study indicated that a metabolic pathway in which ETH is formed as an intermediate does exist in Caco-2 cells. Although the endogenous production of ETH was relatively minute, and probably far too low to have any forensic or medical significance, its possible biochemical interactions (i.e., with some carboxylic acids) deserve future attention.

During incubation, small amounts of ALA, GLA and PRO were detected in both the AC and BLC of GLU- and FRU-supplemented cells, confirming that a bidirectional transport of amino acids took place in this intestinal epithelial cell layer model, as suggested by Sakai et al. [29]. We speculate ALA was produced by the reductive amination of PYR, whose concentration decreased over time.

Since enterocytes express the complete enzymatic machinery required for de novo lipogenesis (DNL) and secretion of triglyceride-rich lipoprotein particles [30], and differences in the metabolism of FRU, mainly the lack of regulation of fructokinase, as compared to GLU may mean it is a more potent precursor for DNL, we verified the existence and entity of this pathway upon supplementation. In our experimental conditions, an active DNL was observed in both GLU- and FRU-supplemented cells. Within the enterocytes, not only saturated but mainly unsaturated fatty acid concentration increased upon supplementation, without significant differences related to the supplemented monosaccharide. Although an increased intestinal expression of lipogenic and lipoprotein genes, including the stearoyl-CoA desaturase 1 gene, was already demonstrated in the mouse intestine and organoids upon FRU supplementation [31], to our knowledge, there are no previous reports about an increased synthesis of unsaturated fatty acids mediated by GLU.

At T8, saturated and monounsaturated fatty acids were detected in the AC. Although the protein-mediated fatty acid uptake system is now believed to be the dominant means by which fatty acids are absorbed by enterocytes, the fatty acid transport across the plasma membrane also occurs by simple, passive diffusion [32], and the absence of lipids in the medium could have favored their efflux in the AC. Fatty acids were detected in the BLC as well, palmitic and stearic acid concentration being higher in FRU- than GLU-supplemented cells. In addition, the total amount of de novo synthetized fatty acids was higher in FRU than GLU-supplemented cells, confirming that FRU specifically stimulates the expression of enterocyte genes for lipid and apolipoprotein synthesis, as already observed in mice [31]. Since a de novo synthesis of linoleic acid is not possible in mammalian cells, we speculate that the small increase of 18:2 n-6 concentration observed in FRU-supplemented cells was related to the retro conversion of longer/more unsaturated n-6 fatty acids. Regarding the more abundant fatty acids, the composition of the Caco-2 cells observed in our study closely resembled data from another study, which detected minor n-6 fatty acids [33]. In our study, the amount of total fatty acids recovered from each well was low, so it was difficult to discriminate the peaks of minor n-6 fatty acids from the background noise.

Over 8 h of culture, the concentration of individual amino acids already included in the AC medium changed to different extents in GLU- and FRU-supplemented cells, suggesting an already reported role of monosaccharides in the regulation of amino acid entry in the circulation. Indeed, a 24%-to-57% higher transport of LEU, ILE, VAL, ALA, PHE, tryptophan (TRP) and histidine (HIS) in the presence of 10 mM FRU than 10 mM GLU was already observed in epithelial cells isolated from the small intestine of rats [34]. Furthermore, in GLU-supplemented cells the total amount (AC + BLC) of amino acids supplemented in the AC medium was similar at T0 and T8 (T0 = 5.58 ± 0.21 mM; T8 = 6.01 ± 0.45 mM; *p* = 0.286), while in FRU-supplemented ones, it significantly decreased over time (T0 = 5.77 ± 0.01 mM; T8 = 4.13 ± 0.46 mM; *p* = 0.0089), suggesting that a remarkable number of amino acids taken up from AC was metabolized. Amino acids are primary carbon sources for DNL, and in vivo tracing studies confirmed dietary amino acids are twice more efficient than GLU in labeling the hepatic acetyl-CoA and fatty acid pool [35]. Thus, in FRU-supplemented cells, the observed increased DNL could be related not only to FRU but also to amino acid metabolization.

## 5. Conclusions

Sugar consumption appears to be causal or contributory in the development of metabolic diseases, and sugar in the form of FRU is clearly the bad guy. Based on the results of 34 stable isotope tracer studies, Sun et al. [36] provided a summary of the quantitative disposal routes of FRU in the body. It was concluded that the mean oxidation rate of dietary FRU is about 45%, and a small percentage of FRU (<1%) is directly converted to plasma TG, while conversion to GLU and LAC occurs to a great extent. The use of Caco-2 cells and NMR plus GC analysis allowed us to further understand the contribution of intestinal cells to FRU systemic metabolization, and the differential effects of GLU and FRU on cell metabolism. Our results confirmed the prominent role of intestinal cells in FRU metabolism and clearance after absorption. Enterocytes shield hepatocytes from FRU exposure, and FRU oxidation seems to occur already in the intestine. Intestinal conversion of FRU to fatty acids was small, although higher than GLU conversion, and enterocytes also contributed to gluconeogenesis. Conversely, enterocytes did not significantly contribute to the systemic conversion of FRU to LAC observed by Sun et al. [36]. Although in future research the use of labeled substrates will be important to confirm GLU and FRU conversion into other metabolites, in the present study, NMR metabolomics provided a powerful tool to study the different impact of the two sugars on the uptake and metabolism of other molecules. In our experimental conditions, differences were small, but they suggest a possible stronger effect in more complex conditions, i.e., after a meal, suggesting a further link between sugar consumption and the onset of metabolic diseases.

The lack of data on expression/activity of transporters/enzymes that could be involved in the observed modifications could be considered a limitation of the present study, which was an untargeted one. It is worth noting that untargeted approaches provide the most appropriate route to detect unexpected changes in metabolite concentrations, and usually precedes targeted ones. In the future, a targeted analysis will be fundamental to further clarify the events occurring in the enterocytes upon GLU and FRU absorption.

It has been well documented that polarized Caco-2 monolayers represent a reliable correlate for studies on the absorption of compounds after oral intake in humans. Several studies have compared Caco-2 permeability coefficients with absorption data in humans and found a high correlation [10]. Evidently, the data analysis of experiments in the Caco-2 cell model, as well as in vivo experiments on animal models, cannot be directly compared with the human situation, but findings from cell culture models can be considered as tracers to facilitate and reduce the complexity of human studies.

## Figures and Tables

**Table 1:** Fatty acid methyl esters (FAME) content in Caco-2 cells at T0 and T8.

		GLU-Supplemented	FRU-Supplemented	ANOVA
FAME	T0	T8		
14:0	3.2 ± 0.3 ^b^	4.0 ± 0.3 ^a^	4.7 ± 0.3 ^a^	*p* = 0.001
16:0	40.7 ± 3.5 ^b^	47.5 ± 6.3 ^ab^	55.0 ± 8.5 ^a^	*p* = 0.0504
16:1 n-7	7.8 ± 0.9 ^b^	9.9 ± 0.7 ^a^	11.4 ± 1.1 ^a^	*p* = 0.0038
18:0	39.3 ± 4.6 ^a^	45.0 ± 7.4 ^a^	52.0 ± 10.1 ^a^	*p* = 0.1457
18:1 n-9	44.8 ± 5.0 ^b^	55.6 ± 3.6 ^a^	61.3 ± 3.5 ^a^	*p* = 0.0037
18:1 n-7	11.6 ± 1.6 ^b^	14.1 ± 0.8 ^ab^	16.0 ± 1.4 ^a^	*p* = 0.0109
18:2 n-6	1.6 ± 0.3 ^b^	1.9 ± 0.1 ^ab^	2.1 ± 0.1 ^a^	*p* = 0.0446
18:3 n-3	2.8 ± 0.2 ^a^	2.7 ± 1.2 ^a^	3.8 ± 0.4 ^a^	*p* = 0.1584
20:4 n-6	3.7 ± 0.6 ^b^	4.3 ± 0.2 ^ab^	4.8 ± 0.3 ^a^	*p* = 0.0366
20:5 n-3	0.7 ± 0.8 ^a^	1.2 ± 0.2 ^a^	1.3 ± 0.2 ^a^	*p* = 0.3442
22:6 n-3	2.7 ± 0.5 ^a^	3.7 ± 0.7 ^a^	3.8 ± 0.6 ^a^	*p* = 0.1298
Total	158.9 ± 11.3 ^b^	189.7 ± 15.5 ^a^	216.0 ± 10.8 ^a^	*p* = 0.0017

Data are expressed as μg/well and are means ± SD of at least 3 samples from independent experiments. Statistical analysis was by one-way ANOVA with Tukey’s post hoc test. Different letters in the same row indicate significant difference (at least *p* < 0.05). GLU: glucose; FRU: fructose.

**Table 2:** Fatty acid methyl esters (FAME) content in the AC and BLC at T8.

	GLU-Supplemented		FRU-Supplemented	
FAME	AC	BLC	AC	BLC
14:0	0.5 ± 0.6	0.23 ± 0.06	0.50 ± 0.13	0.32 ± 0.05
16:0	11.8 ± 2.8	5.80 ± 0.91	13.58 ± 6.19	7.40 ± 0.25 *
18:0	11.1 ± 2.5	5.31 ± 0.81	13.95 ± 5.45	7.13 ± 0.19 *
18:1 n-9	4.3 ± 3.9	0.0 ± 0.0	3.86 ± 3.08	2.39 ± 3.28
Total	27.76 ± 6.12	11.34 ± 1.76	31.89 ± 9.27	17.24 ± 3.61

Data are expressed as μg/well and are means ± SD of at least 3 samples from independent experiments. Statistical analysis was by an unpaired *t*-test to evaluate differences between and GLU and FRU-supplemented cells at T8 (* *p* at least < 0.05). GLU: glucose; FRU: fructose; AC: apical chamber; BLC: basolateral chamber.

## Data Availability

No applicable.

## Supplementary Materials

The following are available online at https://www.mdpi.com/article/10.3390/nu14030517/s1. Figure S1: Time course of SUC diffusion from the apical to the basolateral chamber in SUC-supplemented cells; Figure S2: GLU and FRU concentration in apical and basolateral chambers of SUC-supplemented cells at different time points; Figure S3: ETH, LAC, and PYR concentrations in apical and basolateral chambers of SUC-supplemented cells at different time points; Figure S4: ALA, PRO, and GLA concentrations in apical and basolateral chambers of SUC-supplemented cells at different time points; Figure S5: PHE, LEU, ILE, VAL, MET, and TYR concentrations in apical and basolateral chambers of SUC-supplemented cells at different time points; Figure S6: THR, LYS, ARG, SER, and GLN concentrations in apical and basolateral chambers of SUC-supplemented cells at different time points; Figure S7: PHE, LEU, ILE, VAL, MET, and TYR concentrations in apical and basolateral chambers of GLU- and FRU-supplemented cells at different time points; Figure S8: THR, LYS, ARG, SER, and GLN concentrations in apical and basolateral chambers of GLU- and FRU-supplemented cells at different time points.
