# Supplementary material for: Insight on Glucose and Fructose Absorption and Relevance in the Enterocyte Milieu
Source: Nutrients. 2022 Jan 25;14(3):517. doi: 10.3390/nu14030517 (PMC8839622; doi:10.3390/nu14030517)
Supplement: Supplementary file 1 [file nutrients-14-00517-s001.zip › Chiarello et al_Supplementary Figure S1 Revised.pdf]

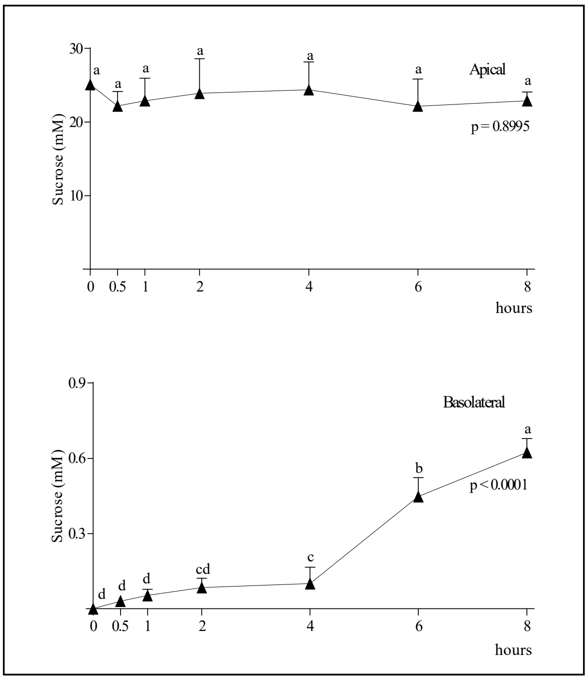


**Figure S1.** Time course of SUC diffusion from the apical to the basolateral chamber in SUC- supplemented cells. SUC concentration (mM) at different time points is indicated by black triangle. Data are means ± SD of at least 3 samples from independent experiments. Statistical analysis was by the one-way ANOVA with Tukey's post-hoc test to compare SUC concentration at different time points (different letters indicate significant differences).
