# Supplementary material for: Insight on Glucose and Fructose Absorption and Relevance in the Enterocyte Milieu
Source: Nutrients. 2022 Jan 25;14(3):517. doi: 10.3390/nu14030517 (PMC8839622; doi:10.3390/nu14030517)
Supplement: Supplementary file 1 [file nutrients-14-00517-s001.zip › Chiarello et al_Supplementary Figure S3 Revised.pdf]

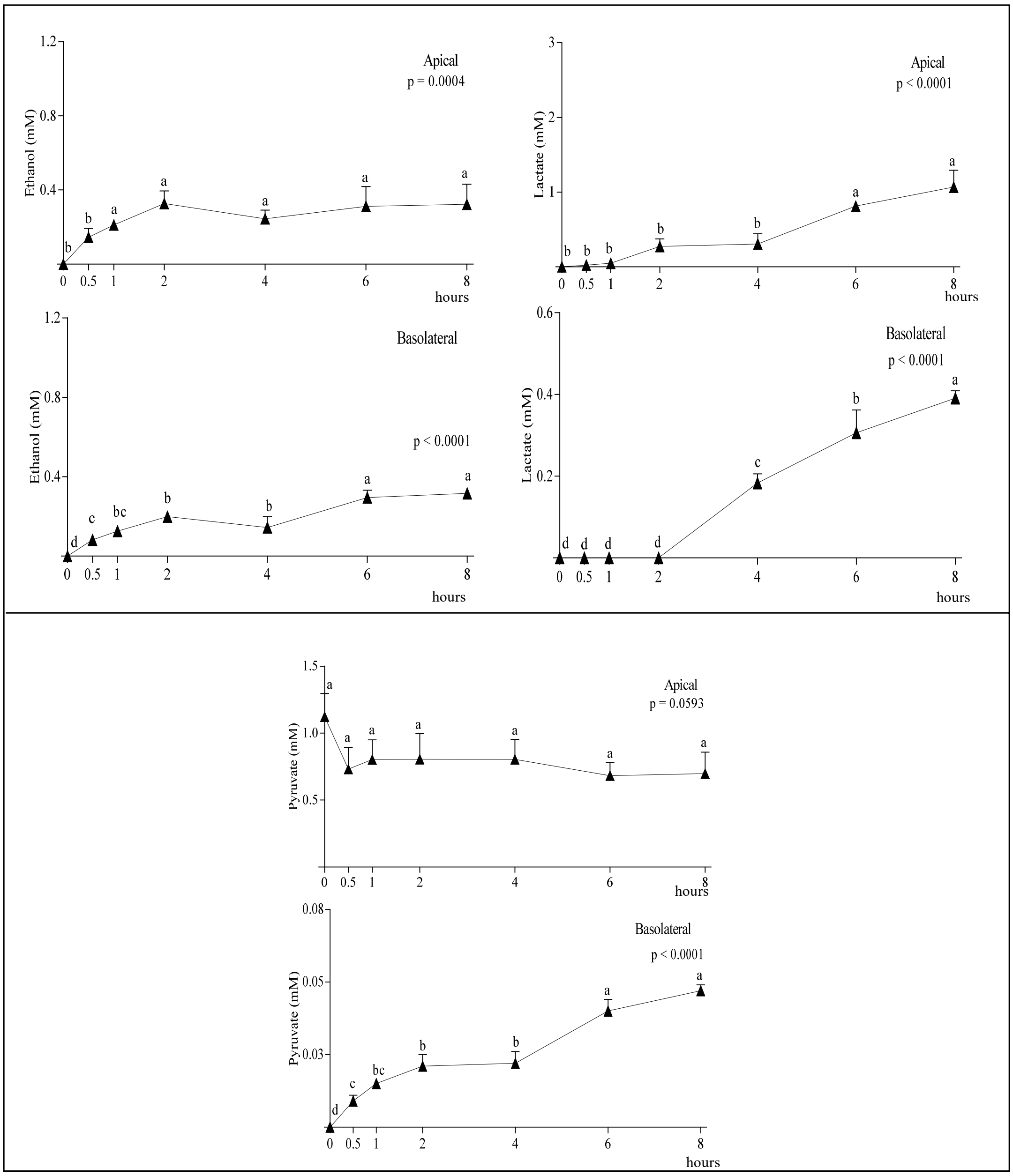


**Figure S3.** ETH, LAC, PYR concentration in apical and basolateral chambers at different time points in SUC-supplemented cells. Data are means ± SD of at least 3 samples from independent experiments. Statistical analysis was by the one-way ANOVA with Tukey's post-hoc test to compare metabolite concentration at different time points (different letters indicate significant differences).
