# Supplementary material for: Insight on Glucose and Fructose Absorption and Relevance in the Enterocyte Milieu
Source: Nutrients. 2022 Jan 25;14(3):517. doi: 10.3390/nu14030517 (PMC8839622; doi:10.3390/nu14030517)
Supplement: Supplementary file 1 [file nutrients-14-00517-s001.zip › Chiarello et al_Supplementary Figure S5 Revised.pdf]

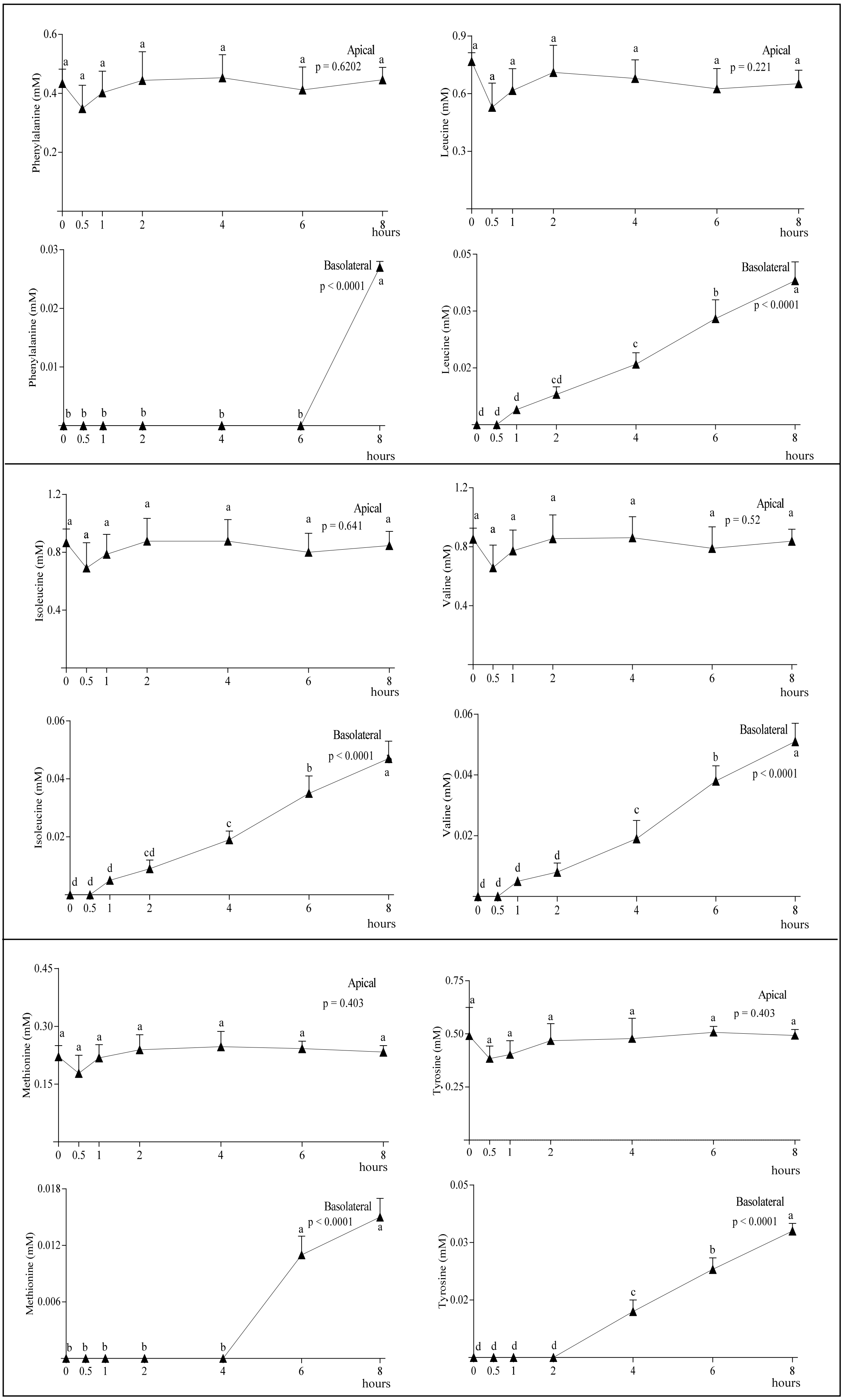
Phenylalanine (mM)
Apical
p = 0.6202
Basolateral
p < 0.0001
Leucine (mM)
Apical
p = 0.221
Basolateral
p < 0.0001
Isoleucine (mM)
Apical
p = 0.641
Basolateral
p < 0.0001
Valine (mM)
Apical
p = 0.52
Basolateral
p < 0.0001
Methionine (mM)
Apical
p = 0.403
Basolateral
p < 0.0001
Tyrosine (mM)
Apical
p = 0.403
Basolateral
p < 0.0001
hours
0 0.5 1 2 4 6 8

Figure S5. PHE, LEU, ILE, VAL, MET, TYR concentration in apical and basolateral chambers at different time points in SUC-supplemented cells. Data are means ± SD of at least 3 samples from independent experiments. Statistical analysis was by the one-way ANOVA with Tukey's post-hoc test to compare metabolite concentration at different time points (different letters indicate significant differences).
