# Supplementary material for: Insight on Glucose and Fructose Absorption and Relevance in the Enterocyte Milieu
Source: Nutrients. 2022 Jan 25;14(3):517. doi: 10.3390/nu14030517 (PMC8839622; doi:10.3390/nu14030517)
Supplement: Supplementary file 1 [file nutrients-14-00517-s001.zip › Chiarello et al_Supplementary Figure S6 Revised.pdf]

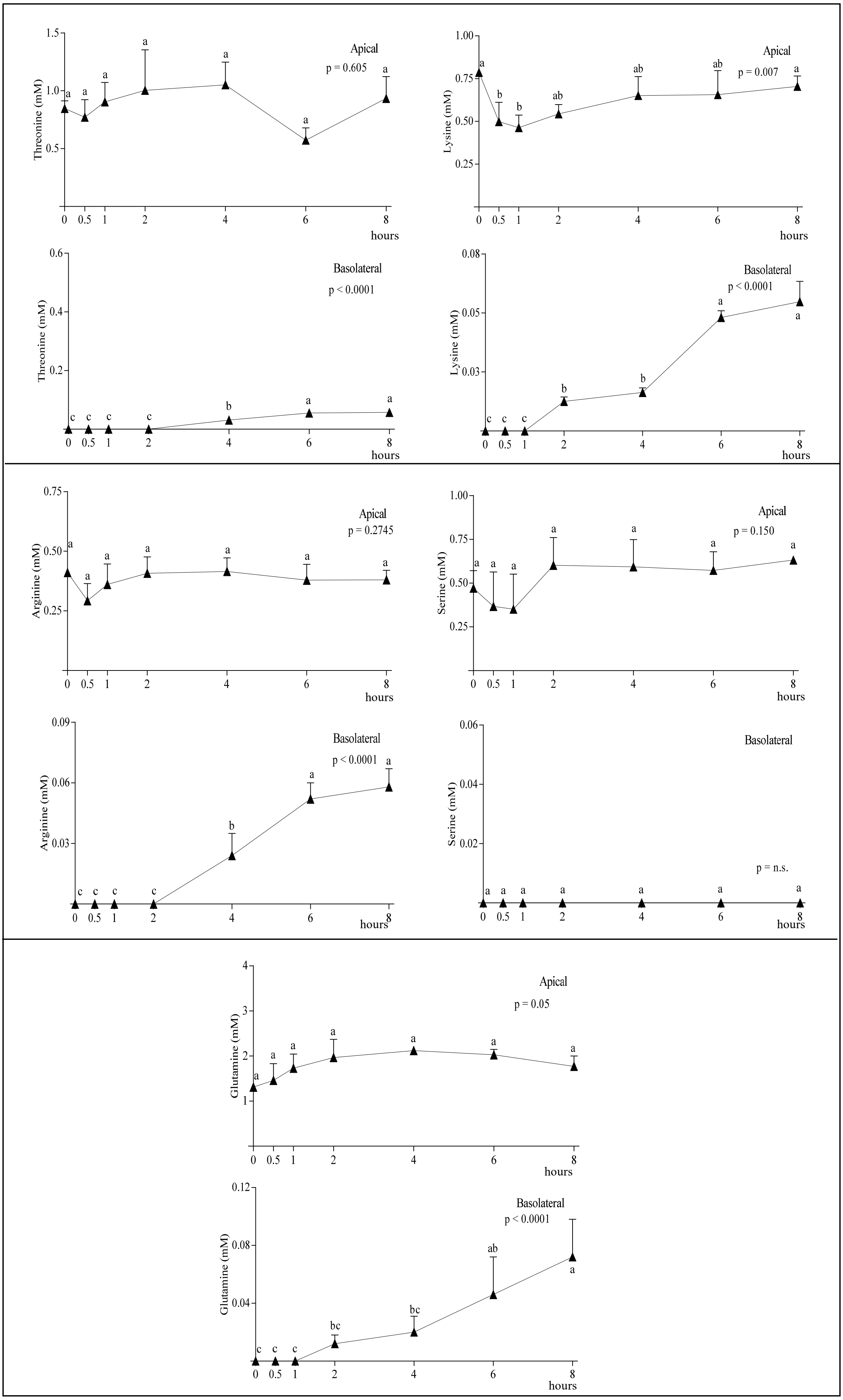

Threonine (mM)
Apical
p = 0.605
hours
Lysine (mM)
Apical
p = 0.007
hours
Threonine (mM)
Basolateral
p < 0.0001
hours
Lysine (mM)
Basolateral
p < 0.0001
hours
Arginine (mM)
Apical
p = 0.2745
hours
Serine (mM)
Apical
p = 0.150
hours
Arginine (mM)
Basolateral
p < 0.0001
hours
Serine (mM)
Basolateral
p = n.s.
hours
Glutamine (mM)
Apical
p = 0.05
hours
Glutamine (mM)
Basolateral
p < 0.0001
hours

Figure S6. THR, LYS, ARG, SER, GLN concentration in apical and basolateral chambers at different time points in SUC-supplemented cells. Data are means ± SD of at least 3 samples from independent experiments. Statistical analysis was by the one-way ANOVA with Tukey's post-hoc test to compare metabolite concentration at different time points (different letters indicate significant differences).
