# Supplementary material for: Insight on Glucose and Fructose Absorption and Relevance in the Enterocyte Milieu
Source: Nutrients. 2022 Jan 25;14(3):517. doi: 10.3390/nu14030517 (PMC8839622; doi:10.3390/nu14030517)
Supplement: Supplementary file 1 [file nutrients-14-00517-s001.zip › Chiarello et al_Supplementary Figure S7 Revised.pdf]

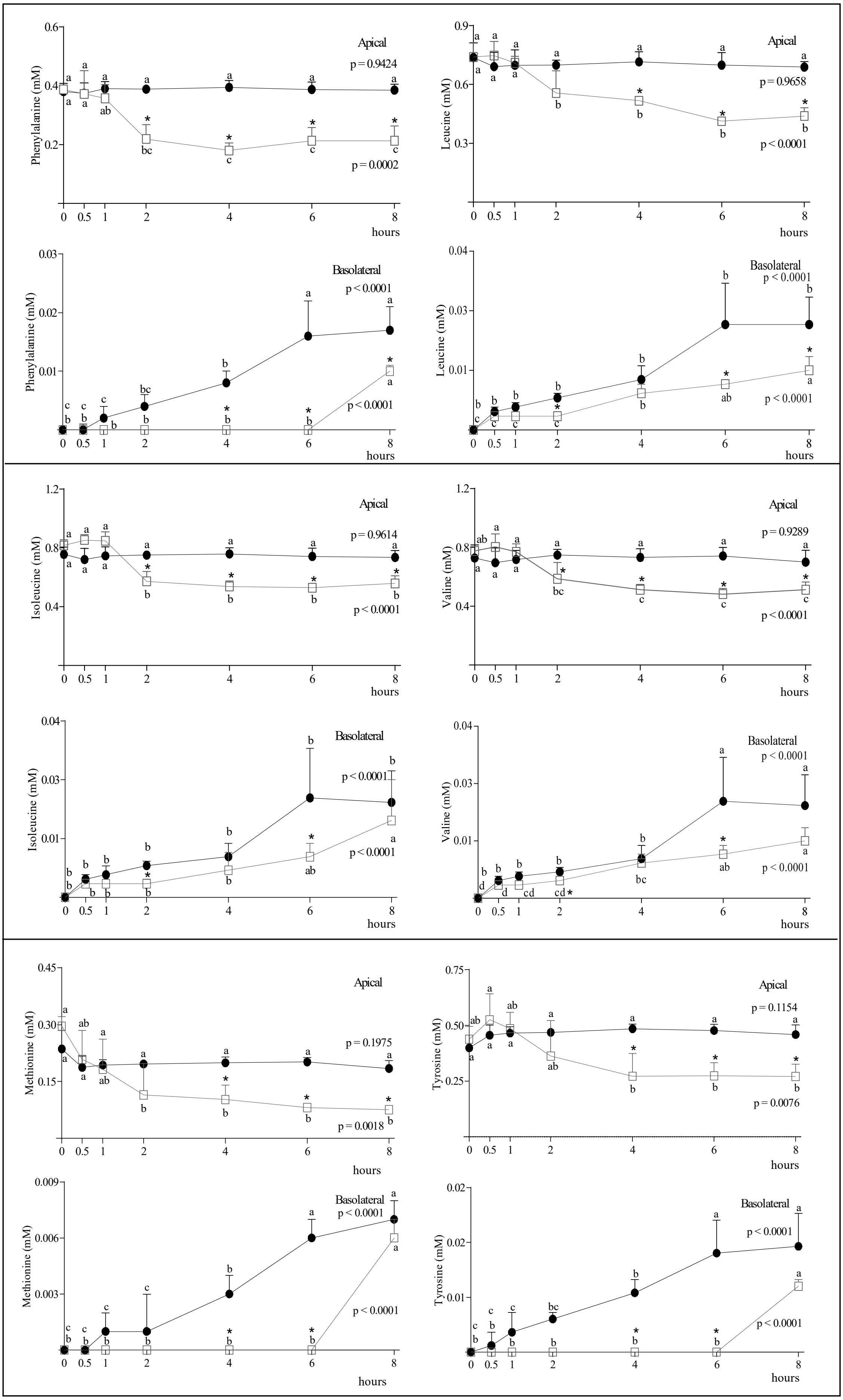

Phenylalanine (mM)
Apical
p = 0.9424
p = 0.0002
hours
Leucine (mM)
Apical
p = 0.9658
p < 0.0001
hours
Phenylalanine (mM)
Basolateral
p < 0.0001
p < 0.0001
hours
Leucine (mM)
Basolateral
p < 0.0001
p < 0.0001
hours
Isoleucine (mM)
Apical
p = 0.9614
p < 0.0001
hours
Valine (mM)
Apical
p = 0.9289
p < 0.0001
hours
Isoleucine (mM)
Basolateral
p < 0.0001
p < 0.0001
hours
Valine (mM)
Basolateral
p < 0.0001
p < 0.0001
hours
Methionine (mM)
Apical
p = 0.1975
p = 0.0018
hours
Tyrosine (mM)
Apical
p = 0.1154
p = 0.0076
hours
Methionine (mM)
Basolateral
p < 0.0001
p < 0.0001
hours
Tyrosine (mM)
Basolateral
p < 0.0001
p < 0.0001
hours

Figure S7. PHE, LEU, ILE, VAL, MET, TYR concentration in apical and basolateral chambers at different time points. Aminoacid concentration at different time points is indicated by black circles (GLU-supplemented cells) or white squares (FRU-supplemented cells). Statistical analysis was by the one-way ANOVA with Tukey's post-hoc test to compare the metabolites concentration at different time points (different letters indicate significant differences) and by the Student's t-test to evaluate differences between GLU and FRU-supplemented cells at each time point (* at least $p<0.05$).
