# Supplementary material for: Insight on Glucose and Fructose Absorption and Relevance in the Enterocyte Milieu
Source: Nutrients. 2022 Jan 25;14(3):517. doi: 10.3390/nu14030517 (PMC8839622; doi:10.3390/nu14030517)
Supplement: Supplementary file 1 [file nutrients-14-00517-s001.zip › Chiarello et al_Supplementary Figure S8 Revised.pdf]

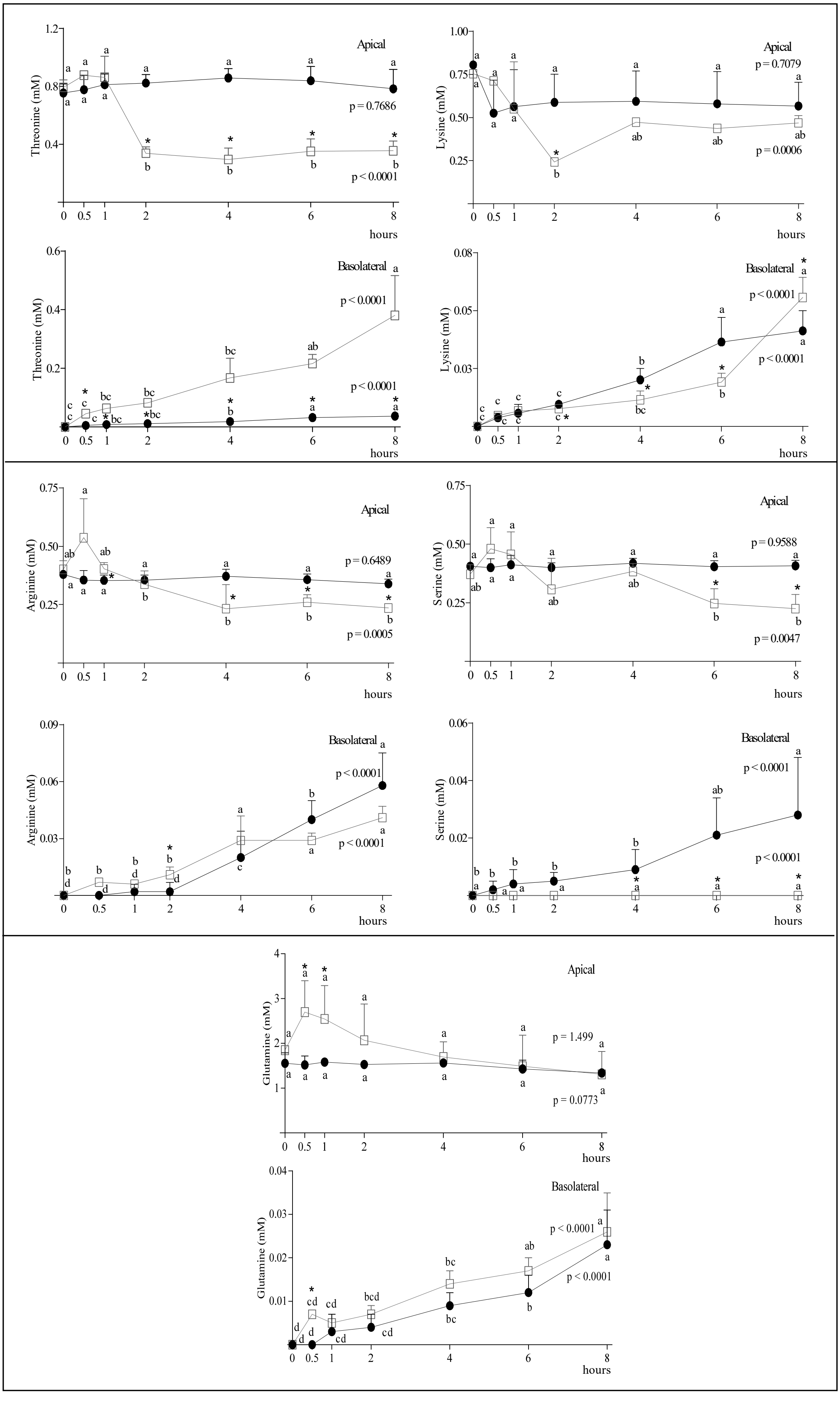

Apical
Threonine (mM)
p = 0.7686
p < 0.0001
hours
Apical
Lysine (mM)
p = 0.7079
p = 0.0006
hours
Basolateral
Threonine (mM)
p < 0.0001
p < 0.0001
hours
Basolateral
Lysine (mM)
p < 0.0001
p < 0.0001
hours
Apical
Arginine (mM)
p = 0.6489
p = 0.0005
hours
Apical
Serine (mM)
p = 0.9588
p = 0.0047
hours
Basolateral
Arginine (mM)
p < 0.0001
p < 0.0001
hours
Basolateral
Serine (mM)
p < 0.0001
p < 0.0001
hours
Apical
Glutamine (mM)
p = 1.499
p = 0.0773
hours
Basolateral
Glutamine (mM)
p < 0.0001
p < 0.0001
hours

Figure S8. THR, LYS, ARG, SER, GLN concentration in apical and basolateral chambers at different time points. Aminoacid concentration at different time points is indicated by black circles (GLU-supplemented cells) or white squares (FRU-supplemented cells). Statistical analysis was by the one-way ANOVA with Tukey's post-hoc test to compare the metabolites concentration at different time points (different letters indicate significant differences) and by the Student's t-test to evaluate differences between GLU and FRU-supplemented cells at each time point (* at least $p<0.05$).
